# Short-Term Total Sleep-Deprivation Impairs Contextual Fear Memory, and Contextual Fear-Conditioning Reduces REM Sleep in Moderately Anxious Swiss Mice

**DOI:** 10.3389/fnbeh.2017.00239

**Published:** 2017-11-29

**Authors:** Munazah F. Qureshi, Sushil K. Jha

**Affiliations:** Sleep Research Laboratory, School of Life Sciences, Jawaharlal Nehru University, New Delhi, India

**Keywords:** anxiety, fear-conditioning, hippocampus, NREM sleep and sleep-deprivation

## Abstract

The conditioning tasks have been widely used to model fear and anxiety and to study their association with sleep. Many reports suggest that sleep plays a vital role in the consolidation of fear memory. Studies have also demonstrated that fear-conditioning influences sleep differently in mice strains having a low or high anxiety level. It is, therefore, necessary to know, how sleep influences fear-conditioning and how fear-conditioning induces changes in sleep architecture in moderate anxious strains. We have used Swiss mice, a moderate anxious strain, to study the effects of: (i) sleep deprivation on contextual fear conditioned memory, and also (ii) contextual fear conditioning on sleep architecture. Animals were divided into three groups: (a) non-sleep deprived (NSD); (b) stress control (SC); and (c) sleep-deprived (SD) groups. The SD animals were SD for 5 h soon after training. We found that the NSD and SC animals showed 60.57% and 58.12% freezing on the testing day, while SD animals showed significantly less freezing (17.13% only; *p* < 0.001) on the testing day. Further, we observed that contextual fear-conditioning did not alter the total amount of wakefulness and non-rapid eye movement (NREM) sleep. REM sleep, however, significantly decreased in NSD and SC animals on the training and testing days. Interestingly, REM sleep did not decrease in the SD animals on the testing day. Our results suggest that short-term sleep deprivation impairs fear memory in moderate anxious mice. It also suggests that NREM sleep, but not REM sleep, may have an obligatory role in memory consolidation.

## Introduction

Sleep is believed to play a role in memory consolidation. Several studies have reported that sleep alteration soon after training induces memory deficit. For example, total sleep deprivation impairs consolidation of declarative, procedural and associative memories (Gais et al., 2000; Graves et al., 2003; Walker et al., 2003; Backhaus et al., 2006; Hill et al., 2008; Chowdhury et al., 2011; Kumar and Jha, 2012; Tripathi and Jha, 2016). Similarly, selective rapid eye movement (REM) sleep deprivation impairs the consolidation of spatial memories (Smith and Rose, 1996; Bjorness et al., 2005). Interestingly, sleep induction on demand (by expressing the temperature-gated nonspecific cation channel in the neurons) soon after training, facilitates memory consolidation in flies (Donlea et al., 2011). Thus, the sleeping brain possibly helps to reactivate neural networks and reinforces memory retention (Jha et al., 2005b; Rasch et al., 2007). Sleep can also play a role in synaptic renormalization. Learning-mediated up-scaled synaptic potentiation in the brain during wakefulness is renormalized during sleep for the homeostatic balance (Bushey et al., 2011). All these suggest that sleep serves a facilitatory role in memory strengthening and stabilization. It, however, remains an intriguing question that if one learns more, would there be a more demand for sleep?

Studies suggest that total sleep or a specific sleep state “REM sleep” or “non-REM (NREM) sleep” or both increase after learning a new task (Smith and Rose, 1996; Walker and Stickgold, 2004; Fogel et al., 2007; Hellman and Abel, 2007; Kumar and Jha, 2012). Some other studies suggest no change in sleep amount *per se*, but instead, they report some changes in its electrophysiological correlates only after learning (Datta, 2000; Gais et al., 2002; Huber et al., 2004; Eschenko et al., 2008). Furthermore, ambiguity persists in learning-dependent sleep demand as sleep architecture alters differently among different strains after learning (Sanford et al., 2003a,b; Tang et al., 2005). For example, NREM sleep increases in less anxious and decreases in highly anxious mice strains after fear conditioning (Sanford et al., 2003b). On the other hand, NREM sleep does not change if the animals are fear-conditioned with a single tone-shock paired presentation (Sanford et al., 2003a). It, however, alters if the animals are fear-conditioned with multiple tone-shock paired stimuli (Sanford et al., 2003a). REM sleep, although, plays an essential role in the consolidation of emotional memory (Wagner et al., 2001), but it remains suppressed after fear conditioning (Jha et al., 2005a; Pawlyk et al., 2005; Kumar and Jha, 2012, 2017). Since the highly anxious animals demonstrate more profound fear- and anxiety-related behaviors (Bert et al., 2002), it is not yet clear, if a considerable alteration in sleep patterns after learning can be attributed to the processes of fear-conditioning or susceptibility of the animals to their general anxiety level.

Different strains of mice demonstrate different anxiety levels (Paylor et al., 1994; Falls et al., 1997; O’Leary et al., 2013). For example, C57BL/6J strain demonstrates low anxiousness and anxiety, whereas BALB/c and CB6F1/J mice strains are highly anxious and susceptible to anxiety (Griebel et al., 2000; Sanford et al., 2003b). These mice strains have been previously used to study the influence of fear-conditioning on sleep architecture. It was found that fear-conditioning influences sleep differently in these mice strains (Sanford et al., 2003a,b). Further, the less anxious mice strain, C57BL/6J, has been used to study the effects of sleep deprivation in the consolidation of fear memory (Graves et al., 2003; Vecsey et al., 2009). The effects of sleep alteration on contextual fear memory and the influences of contextual fear memory on sleep architecture in moderate-anxious mice are, however, not known. Swiss mice demonstrate moderate reactivity, anxiousness and anxiety behavior in different anxiolytic tasks (Griebel et al., 2000). Hence, we have studied: (a) the effects of short-term sleep deprivation on the consolidation of contextual fear memory: and (b) the influence of contextual fear memory on sleep architecture in Swiss albino mice.

## Materials and Methods

Male Swiss albino mice weighing 30–35 g, (2–3 months old) were used in this study. The animals were brought from the University’s Central Laboratory Animal Resources (CLAR) a week before the commencement of experiments to the school’s in-house animal room facility for acclimatization to the new environment. Animals were housed in plastic cages (in groups of two or three) in temperature (23–24)°C and light controlled (12:12 light-dark cycle; lights on at 7:00 AM) conditions. Food and water were given *ad libitum*. All procedures used in this study were approved by the Institutional Animal Ethics Committee (IAEC) of Jawaharlal Nehru University, New Delhi, India (protocol # 08/2013).

We have studied: (a) the effects of sleep deprivation on the consolidation of contextual fear memory and (b) the changes in sleep architecture after contextual fear training and testing. The animals were randomly divided into three groups: non-sleep deprived (NSD; *n* = 8), sleep-deprived (SD; *n* = 6), and stress control (SC; *n* = 5). The same animals were further used to study the influence of contextual fear conditioning on sleep architecture. Animals were first engaged in the contextual fear conditioning procedure between 11:25 AM and 11:30 AM and after that, their sleep-wakefulness (S-W) was recorded for 5 h between 11:30 AM and 4:30 PM. Sleep could not be recorded in three NSD animals because of some procedural problems. Sleep was also recorded during the sleep-deprivation period in the SD animals.

### Surgical Procedures for Polysomnographic Recordings

Mice were surgically prepared for S-W recordings. Surgery was performed under sterile conditions. The animal was anesthetized with isoflurane (2%) using anesthesia face mask. Head was shaved, and the animal was fixed in the stereotaxic instrument. The level of the Bregma and Lambda was brought on the same horizontal plane. The skull skin was disinfected with a betadine swab, a midline incision was made, and the skull was exposed to electrode implantations. Two pairs of small, stainless-steel screw electrodes were implanted on the frontal (anterior-posterior (AP) + 2 mm; lateral (L) 2 mm) and parietal (AP: 2 mm, L: 2 mm; reference from the Bregma) bones to record electroencephalogram (EEG). Three electrodes (flexible insulated wire except at the tip) were implanted in the dorsal neck muscles to record bipolar electromyogram (EMG; third EMG was implanted as an extra safeguard). One screw electrode was fixed laterally in the nasal bone as a reference electrode. The free ends of the EEG, EMG and reference electrodes were then connected to an eight-pin bug-strip miniature connector, which was cemented onto the skull with dental acrylic and finally the skin was sutured. After surgery, the anesthesia face mask was detached, and the animal was taken out from the stereotaxic instrument.

The animal was treated postoperatively with dexamethasone (1.5 mg/Kg, i.p.) for 2–3 days to reduce brain inflammation and Nebasulf powder (antibiotic) to control infection. The animal was also given a painkiller ibuprofen (0.1 ml/day) and multivitamin syrup (0.2 ml) orally for 2 days. The animal was thus allowed 5–6 days to recover from surgery and after that was engaged in experiments.

### Contextual Fear-Conditioning (CxFC) in Mice

**Figure 1:**
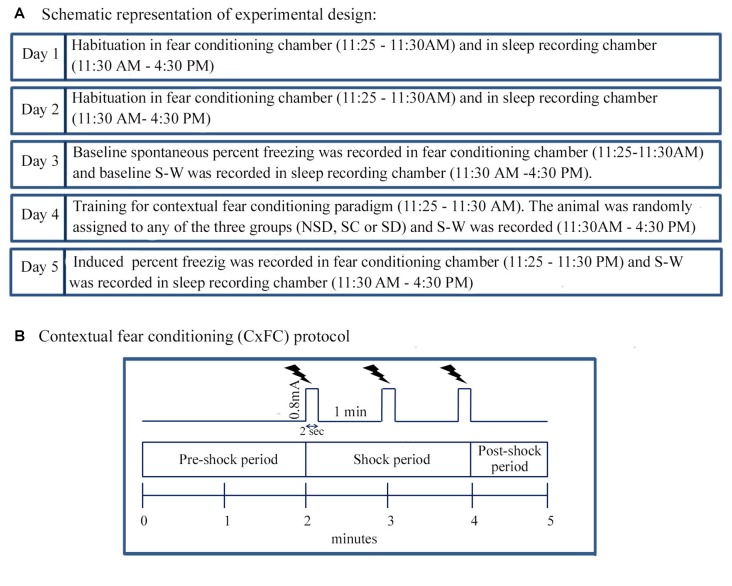
The experimental design and Contextual fear conditioning protocol. **(A)** The animal was habituated in the fear-conditioning chamber between 11:25 AM and 11:30 AM and afterwards in the sleep recording chamber between 11:30 AM and 4:30 PM for 2 days (Day 1 and 2). The animal was also tethered to the sleep recording setup during habituation. On Day 3, the animal was placed in the fear-conditioning chamber, and spontaneous freezing was recorded between 11:25 AM and 11:30 AM. After that, baseline sleep-wakefulness (S-W) was recorded in the sleep recording chamber between 11:30 AM and 4:30 PM. On Day 4, the animal was trained for contextual fear conditioning using standard protocol during the time matched hour of the baseline day. The animal was then randomly assigned to either of the three groups: (a) non-sleep deprived (NSD); (b) stress control (SC); and (c) sleep-deprived (SD) group. S-W was recorded between 11:30 AM and 4:30 PM soon after contextual fear conditioning. On the subsequent day (Day 5), the animal was tested for contextual fear conditioning, and sleep was recorded during time-matched hour of baseline and training days. **(B)** The animal was kept in the conditioning chamber and was allowed to explore the chamber for initial 2 min (pre-shock period). After that, three-foot shocks (current strength: 0.8 mA, duration: 2 s inter-shock interval: 1 min) as an unconditioned stimulus was presented over 2 min period (shock-period). The animal was removed from the conditioning chamber 1 min after the last shock (post-shock period).

Animals were trained for contextual fear conditioning using the standard protocol (Figure 1). Fear-condition training was performed in a shock chamber (Coulbourn Inc., Whitehall, PA, USA). The shock chamber was kept inside a sound and light dampened box (26″ × 24″ × 18″ black box) to minimize external disturbances during experiments. The conditioning chamber was cleaned before and after each use with Colin surface cleaner (Reckitt Benckiser, India).

Before commencing the fear-condition training, the animal was habituated in a neutral chamber for 2 days (Day 1 and 2) for 5 min between 11:25 AM and 11:30 AM (Figure 1A). The chamber was illuminated with 20 Lux light. On Day 3, the animal was placed in the conditioning chamber, and spontaneous freezing behavior was recorded in a computer as baseline using CCTV camera (SenTech, Carrollton, TX, USA) and FreezeFrame software (Coulbourn Instruments, Whitehall, PA, USA). On Day 4, the person who had not handled the animal before (unfamiliar person) brought the animal from the animal colony via a different route and placed the animal in the behavioral chamber. It was done to rule out the contextual reminders related to the familiar person and route. After that, the animal was trained for contextual fear-conditioning in the conditioning chamber with some additional situational reminders. For example, the illumination of the conditioning chamber was increased from 20 Lux to 80 Lux, and 0.6 ml of sandalwood fragrance (Air Wick-Mystic sandal and jasmine, Reckitt Benckiser, India) was added in the bedding.

The protocol in the FreezeFrame software (Coulbourn Instruments, Whitehall, PA, USA) was written in such a way that during the initial 2 min, no shock was delivered (pre-shock period; Figure 1B). The animal was allowed to explore the conditioning chamber during this period. After that, FreezeFrame software triggered the Coulbourn Precision regulated shocker (model # H13–17) to deliver three-foot shocks of 0.8 mA each for a 2 s duration at an interval of 1 min through the grid floor of the shock chamber (shock period; Figure 1B). The animal was removed from the shock chamber 1 min after the training was over (post-shock period; Figure 1B). The induced freezing behavior was recorded over the entire 5 min period, for offline analysis. On the testing day, the animal was brought by the familiar person through the usual route and was placed in the conditioning chamber at the time matched hour of the baseline and training days. The training day’s bedding was used on the testing day (Day 5) for the contextual reminder. The animal was tested for contextual fear conditioning in the same chamber, but no foot shocks were delivered during testing. The induced freezing was recorded during the entire 5 min period.

### Polysomnographic Recording Procedures

S-W was recorded in each mouse on the baseline, CxFC training and testing days between 11:30 AM and 4:30 PM. A transparent sleep recording box (12″ × 10″ × 12″) was placed inside a black plexiglass (48″ × 24″ × 24″) sleep recording chamber. The sleep recording chamber was well ventilated, sound dampened and sufficiently illuminated (20 Lux light illumination). The animal was habituated to the sleep recording chamber for 2 days, during which, they remained tethered with the recording set-up. Food and water bottle were placed in the attached food cup and bottle holder in the sleep recording box. The mouse was tethered to the recording set-up through an eight-core recording cable via a commutator. The EEG and EMG signal strength and quality were viewed and examined on the habituation days in a computer through Spike2 software (Cambridge Electronic Design, Cambridge, UK). The polysomnographic recordings were, however, acquired from the baseline day onwards. EEGs were recorded in two channels, and EMG was recorded in a single channel. Electrophysiological signals were filtered and amplified using 15LT Bipolar Portable Physiodata Amplifier System (Astro-Med, West Warwick, RI, USA). EEG signals were processed with a high-pass filter of 0.1 Hz and a low-pass filter of 40 Hz, while EMG signal was processed with a high-pass filter of 10 Hz and a low-pass filter of 90 Hz digitized at 100 Hz sampling rate. Recordings were acquired in a computer using Spike2 software (Cambridge Electronic Design, Cambridge, UK) and were saved for offline analysis.

### Sleep Deprivation

Sleep deprivation was performed for 5 h (11:30 AM – 4:30 PM) using gentle handling and a motorized “mouse walking wheel” instrument (Fabricated by University Science Instrumentation Center, JNU, New Delhi). The mouse walking wheel instrument has two components: (i) a motorized rotating wheel (transparent plexiglass, with a wheel size of 6 inches and wheel width of 3.5 inches and one DC operated motor); and (ii) the controller unit (for controlling wheel rotation, with maximum obtainable wheel rotation of 11 RPM). The rotating wheel was placed inside the sleep recording chamber to record vigilant state during sleep deprivation. The controller unit was, however, kept outside the recording chamber. It was done to avoid any electrical interference during S-W recording. The animal was kept inside the rotating wheel soon after contextual fear conditioning. The animal was tethered to a commutator through the recording cable (recording cable was taken out from the central portion of the side wall of the wheel), and the motor was switched on to rotate the wheel. The wheel was initially rotated at a slow speed (3–4 RPM), and later, the speed varied between 4 RPM and 8 RPM (but not more than 8 RPM) during sleep deprivation. The rotation was periodically stopped for 4–5 min to allow the mouse to eat food and drink water. The animal was gently handled and kept awake during this period. S-W was recorded during the entire sleep-deprivation period using the same procedure as mentioned above. At the end of the sleep deprivation period, the mouse was taken out and brought back to the animal colony and left undisturbed for next 18 h.

### Stress Control

To account for sleep deprivation associated stress, we used another group of mice (*n* = 5) as SC. We used 400 Lux high intense light as a stressor as has been used previously (Hale et al., 2006; Bouwknecht et al., 2007; Nathiya and Vanisree, 2010; Kumar and Jha, 2012). Rodents avoid bright light, and if kept just for an hour, they exhibit signs of distress and anxiety (Hale et al., 2006; Nathiya and Vanisree, 2010). Furthermore, the level of plasma cortisol increases three-four folds after 30 min exposure to high-intensity light (Ishida et al., 2005). Interestingly, similar three-four folds increase in plasma cortisol level has also been reported after short-term total sleep deprivation (Hairston et al., 2001). Therefore, considering these facts, we chose bright light as a stressor in the SC group. The animal was kept in the sleep recording chamber soon after CxFC and was continuously exposed to high-intensity light for 5 h (11:30 AM – 4:30 PM). S-W was recorded during this period using a similar procedure as was used for the NSD and SD animals.

### Data Analysis

#### Analysis of Freezing Behavior

Freezing behavior was analyzed offline by using FreezeFrame software (Coulbourn Inc, Whitehall, PA, USA). The percent freezing response was calculated for the entire 5 min period and also during 0–2, 2–4 and 4–5 min time interval (corresponding time intervals of the pre-shock, shock, and post-shock periods of the training day) on baseline, training and testing days. Bout length of the motion index was kept at 2 s which means that if the animal remained motionless for 2 s or more, only then it was registered by the software as freezing. The threshold for freezing index was kept at 10%, at which the freezing bout peak falls mostly near zero on the abscissa in the time graph. This threshold allowed the software to count the breathing-associated movement as freezing. Freezing behavior was thus analyzed and calculated (using stringent criteria) by the computer only, without any manual intervention. The average % freezing at all three time intervals and also for the entire 5 min period was calculated. The changes in the freezing response within and between groups on baseline, training and testing days were compared statistically using one-way RM-ANOVA and one-way ANOVA followed by Tukey *post hoc* test, respectively.

#### Analysis of Polysomnographic Records

The Spike2 polysomnographic records were converted into “European Data Format” and were scored offline using “Somnologica Science Software” (Medcare Flaga, Iceland). Records were displayed on a computer in “Somnologica”, and vigilant states were manually scored using 4 s epochs. The low-voltage and high-frequency EEG waves with increased motor activity were analyzed as wake, whereas the high-voltage, low-frequency EEG waves with prominent delta waves (0.5–4 Hz) and decreased motor activity were analyzed as NREM sleep. The low-voltage, high-frequency EEG waves with a prominent theta peak (5–9 Hz) and nuchal muscle atonia were analyzed as REM sleep. The total time spent in wake, NREM and REM sleep was calculated and expressed as the total mean percent of the total recording time (TRT). The changes in different vigilant states were compared statistically between groups using one-way ANOVA followed by Tukey *post hoc* test and within groups using one-way RM-ANOVA.

## Results

### The Effect of Sleep Deprivation on the Consolidation of Contextual Fear Memory

**Figure 2:**
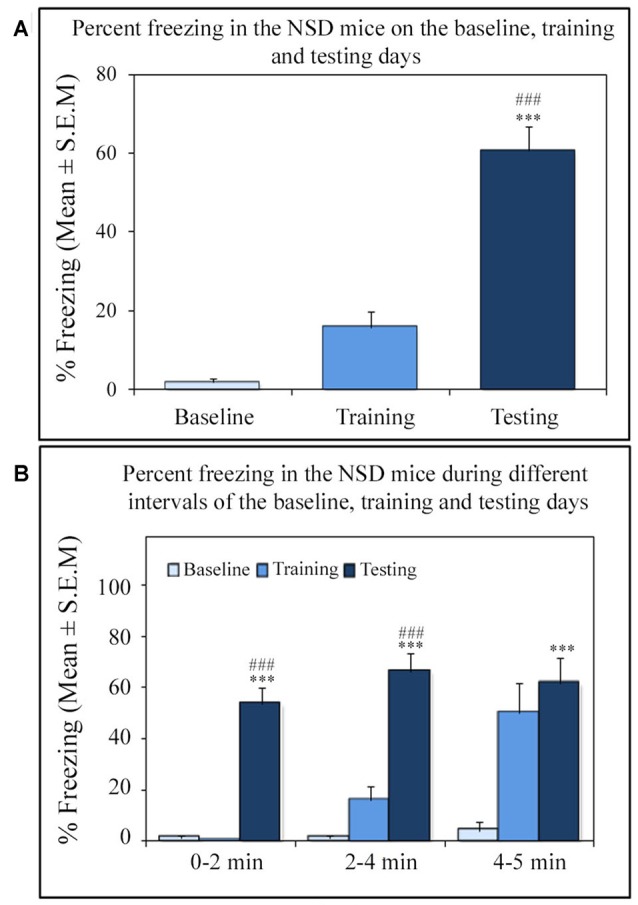
The percent freezing response in Non-SD (NSD) animals on the baseline, training and testing days. **(A)** The NSD mice exhibited a significant increase in percent freezing (*p* < 0.001; *F*_(2,23)_ = 60.64) on the testing day (compared to baseline day: Tukey *p* < 0.001; compared to training day: Tukey *p* < 0.001). **(B)** The percent freezing significantly increased on the testing day during the 0–2 min (compared to baseline day: Tukey *p* < 0.001; compared to training day: Tukey *p* < 0.001), 2–4 min (compared to baseline day: Tukey *p* < 0.001; compared to training day: Tukey *p* < 0.001), and 4–5 min (compared to baseline day: Tukey *p* < 0.001) time periods (the corresponding times of pre-shock, shock and post-shock periods of the training day). ***p < 0.001 (compared to baseline day); ^###^*p* < 0.001 (compared to training day).

**Figure 3:**
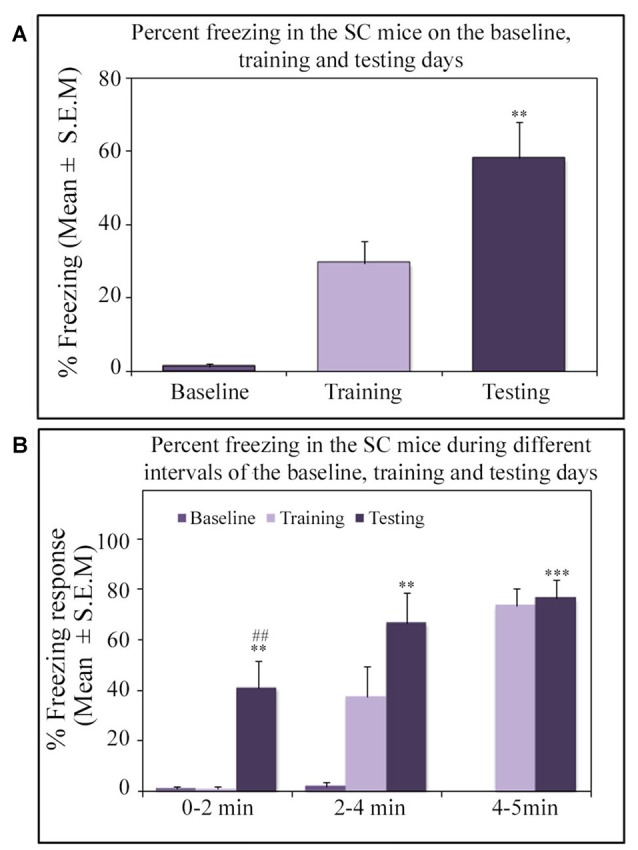
The percent freezing response in the SC mice on the baseline, training and testing days. **(A)** The SC mice exhibited a significant increase in percent freezing (*p* < 0.01, *F*_(2,14)_ = 13.14) on the testing day (compared to baseline day: Tukey *p* < 0.01). **(B)** The percent freezing significantly increased on the testing day during the 0–2 min (compared to baseline day: Tukey *p* < 0.01; compared to training day: Tukey *p* < 0.01), 2–4 min (compared to baseline day: Tukey *p* < 0.01), and 4–5 min (compared to baseline day: Tukey *p* < 0.001) time periods (the corresponding times of pre-shock, shock and post-shock periods of the training day). **p < 0.01 and ***p < 0.001 (compared to baseline day); ^##^*p* < 0.01 (compared to training day).

**Figure 4:**
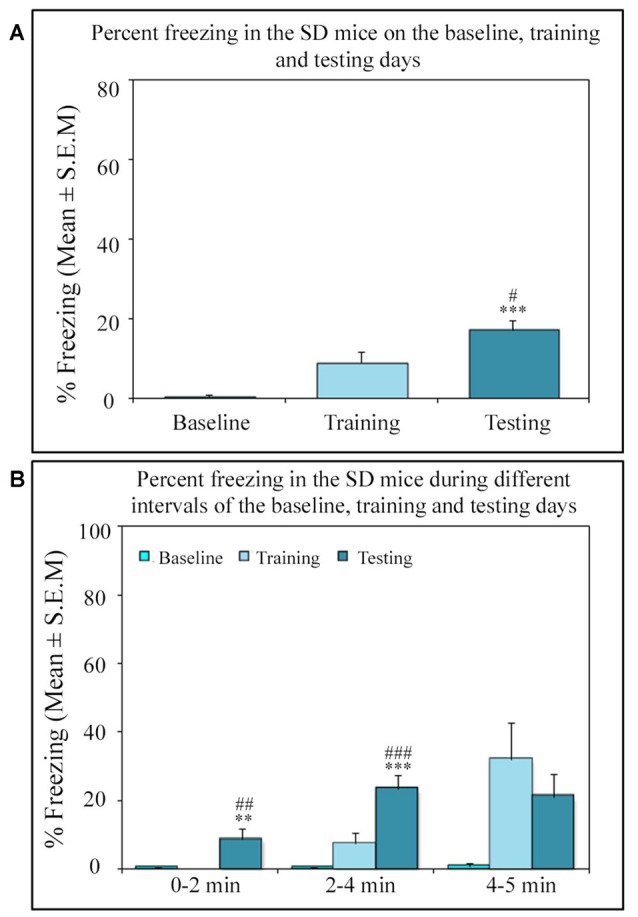
The percent freezing response in the SD mice on the baseline, training and testing days. **(A)** The SD mice also exhibited a significant increase in percent freezing (*p* < 0.001, *F*_(2,17)_ = 22.16) on the testing day (compared to baseline day: Tukey *p* < 0.001 and compared training day: Tukey *p* < 0.05). **(B)** The percent freezing significantly increased on the testing day only during the 0–2 min (compared to baseline day: Tukey *p* < 0.01; compared to training day: Tukey *p* < 0.01) and 2–4 min (compared to baseline day: Tukey *p* < 0.001; compared to training day: Tukey *p* < 0.001) time periods (the corresponding times of pre-shock, shock and post-shock periods of the training day). **p < 0.01 and ***p < 0.001 (compared to baseline day); ^#^*p* < 0.05, ^##^*p* < 0.01 and ^###^*p* < 0.001 (compared to training day).

Similar to other mice strains, Swiss mice also exhibited robust freezing response on the testing day. The NSD animals (*n* = 8) showed significantly high freezing response (60.57%) during the 5 min testing period (*p* < 0.001; *F*_(2,23)_ = 60.64; *Cohen’s d* = 4.65; power = 1.0 at alpha value 0.05; compared to the baseline day: Tukey *p* < 0.001 and compared to the training day: Tukey *p* < 0.001; Figure 2A). The freezing response was significantly high during 0–2 min interval (*p* < 0.001, *F*_(2,23)_ = 67.95), 2–4 min interval (*p* < 0.001, *F*_(2,23)_ = 43.63) and 4–5 min interval (*p* < 0.001, *F*_(2,23)_ = 15.60) on the testing day compared to the time matched interval on baseline and training days (Figure 2B).The SC animals (*n* = 5) also demonstrated a similar freezing behavior on the testing day. The SC animals showed a significant increased freezing response on the testing day (58.12%; *p* < 0.01; *F*_(2,14)_ = 13.15; *Cohen’s d* = 3.62; power = 0.96 at alpha value 0.05) compared to the baseline (Tukey *p* < 0.01; Figure 3A). The SC animals exhibited a significant increased freezing response during 0–2 min interval (*p* < 0.01, *F*_(2,14)_ = 14.94), 2–4 min interval (*p* < 0.01, *F*_(2,14)_ = 7.40) and 4–5 min interval (*p* < 0.001, *F*_(2,14)_ = 38.62) on the testing day compared to the time matched intervals on baseline and training days (Figure 3B). The SD animals (*n* = 6) also exhibited increased freezing behavior (17.13%; *p* < 0.001; *F*_(2,17)_ = 22.16; *Cohen’s d* = 4.40; power = 1.00 at alpha value 0.05; compared to the baseline (Tukey *p* < 0.001) and compared to the training day: Tukey *p* < 0.05) on the testing day (Figure 4A). The SD animals exhibited a significantly increased freezing response only during 0–2 min interval (*p* < 0.01, *F*_(2,17)_ = 9.01) and 2–4 interval (*p* < 0.001, *F*_(2,17)_ = 28.15) on the testing day compared to baseline and training days (Figure 4B). However, the change in the freezing response during the 4–5 min interval was statistically not significant.

**Figure 5:**
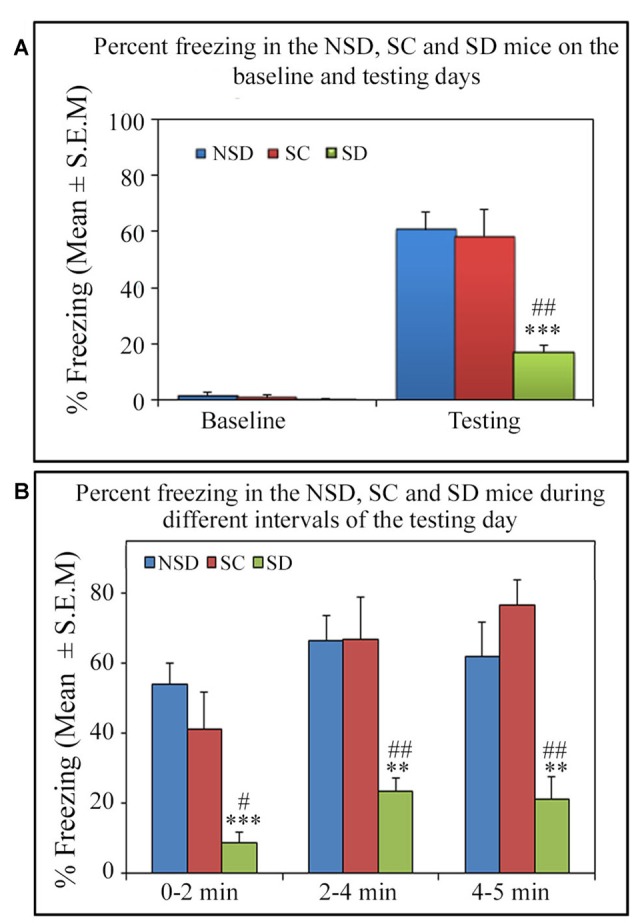
The comparative changes in percent freezing response in the NSD, SC and SD animals. **(A)** The NSD, SC and SD animals showed a comparable freezing response on the baseline day. The percent freezing in the SD animals was significantly less (*p* < 0.001, *F*_(2,18)_ = 13.82) compared to the NSD (Tukey *p* < 0.001) and SC (Tukey *p* < 0.01) animals. **(B)** In the SD animals, the precent freezing was significantly less on the testing day during the 0–2 min (compared to NSD group: Tukey *p* < 0.001; compared to SC group: Tukey *p* < 0.05), 2–4 min (compared to NSD group: Tukey *p* < 0.01; compared to SC group: Tukey *p* < 0.01) and 4–5 min (compared to NSD group: Tukey *p* < 0.01; compared to SC group: Tukey *p* < 0.01) time periods (the corresponding times of pre-shock, shock and post-shock periods of the training day). **p < 0.01 and ***p < 0.001 (compared to NSD group); ^#^*p* < 0.05 and ^##^*p* < 0.01 (compared to SC group).

On the testing day, the NSD and SC animals exhibited a robust freezing response, but surprisingly, the SD animals showed significantly less freezing (*p* < 0.001, *F*_(2,18)_ = 13.82; *Cohen’s d* = 2.59; power = 0.99 at alpha value 0.05) compared to the NSD (Tukey *p* < 0.001) and SC (Tukey *p* < 0.01) groups. The SD animals showed 71.7% (Tukey *p* < 0.001) and 70.52% (Tukey *p* < 0.01) less freezing on the testing day compared to NSD and SC animals, respectively. Nevertheless, the NSD and SC animals showed a comparable freezing response on the testing day (Figure 5A). We also compared the percent freezing response during 0–2 min, 2–4 min and 4–5 intervals in the animals of NSD, SC and SD groups on the testing day. The NSD and SC animals showed a comparable freezing response at every time point. The SD animals, however, showed significantly less freezing response compared to the NSD and SC animals across all intervals (0–2 min interval: *p* < 0.001; *F*_(2,18)_ = 11.98; compared to the NSD: Tukey *p* < 0.001; compared to the SC Tukey *p* < 0.05), (2–4 min interval: *p* < 0.01; *F*_(2,18)_ = 9.79; compared to NSD: Tukey *p* < 0.01; compared to SC Tukey *p* < 0.01), (4–5 min interval: *p* < 0.001; *F*_(2,18)_ = 10.04; compared to NSD: Tukey *p* < 0.01; compared to SC Tukey *p* < 0.01; Figure 5B). These results suggest that contextual fear memory was consolidated in the NSD and SC groups, while it was impaired in the SD group.

### The Changes in Sleep Architecture after Contextual Fear Training and Testing

**Figure 6:**
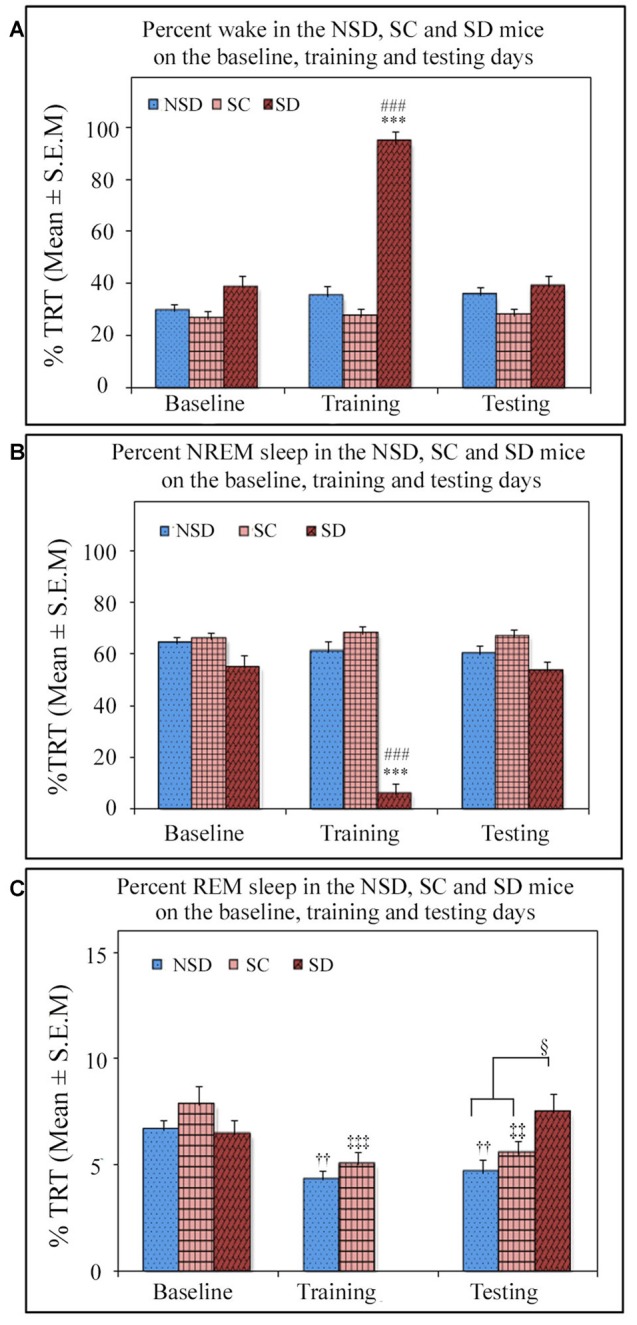
The comparative changes in percent amount of wake, NREM and REM sleep (out of total recording time (TRT)) after contextual fear conditioning in the NSD, SC and SD mice on the baseline, training and testing days. **(A)** The percent wake amount was comparable between groups on the baseline, training and testing days except on the training day in the SD mice. The SD mice were SD on the training day, and they were almost 94% time awake during the deprivation period (*p* < 0.001, *F*_(2,14)_ = 91.645; compared to NSD (Tukey *p* < 0.001) and SC (Tukey *p* < 0.001) animals). **(B)** The percent NREM sleep amount was also comparable between groups except on the training day in the SD mice. The SD animals exhibited significantly less (6%) NREM sleep (*p* < 0.001, *F*_(2,14)_ = 80.436; compared to NSD (Tukey *p* < 0.001) and SC (Tukey *p* < 0.001) animals) during the sleep-deprivation period. **(C)** The percent REM sleep was comparable between groups on the baseline day. However, REM sleep significantly decreased on the training and testing days in the NSD and SC mice (in NSD mice: the training day Tukey *p* < 0.01 and testing day Tukey *p* < 0.01 (compared to the baseline day) and in SC mice: the training day Tukey *p* < 0.001, and testing day Tukey *p* < 0.01 (compared to the baseline day)). There was no rapid eye movement (REM) sleep in the SD mice on the training day as they were sleep deprived, but interestingly they showed no change in REM sleep expression on the testing day compared to the baseline day. The SD mice, however, showed significantly more REM sleep (*p* < 0.05, *F*_(2,14)_ = 4.06) on the testing day compared to the NSD and SC mice (compared to NSD Tukey *p* < 0.05). ***p < 0.001(compared to the NSD group); ^###^*p* < 0.001 (compared to the SC group); ^††^*p* < 0.01 (compared to the NSD baseline); ^‡‡^*p* < 0.01, ^‡‡‡^*p* < 0.001 (compared to the SC baseline) and ^§^*p* < 0.05 (compared to NSD and SC groups on the testing day).

We further studied the influence of contextual fear conditioning on sleep architecture in the memory consolidated-groups: (a) NSD (*n* = 5) and (b) SC (*n* = 5) groups and memory-impaired (SD) group (*n* = 5). The contextual fear conditioning did not alter wakefulness and NREM sleep amount in any of these groups (Figures 6A,B). However, REM sleep significantly decreased on the training and testing days compared to the baseline day in memory-consolidated groups (NSD and SC animals; Figure 6C). Interestingly, REM sleep did not change on the testing day in the memory-impaired group (SD group; Figure 6C). The NSD animals showed 34.8% and 30% less REM sleep (*p* < 0.01, *F*_(2,14)_ = 14.28; *Cohen’s d* = 2.9; power = 0.98 at alpha value 0.05) on the training (Tukey *p* < 0.01) and testing days (Tukey *p* < 0.01) compared to the baseline day, respectively. The decrease in the REM sleep amount was due to significantly less number of REM sleep episodes (*p* < 0.05, *F*_(2,14)_ = 6.093; *Cohen’s d* = 1.81; power = 0.65 at alpha value 0.05) on the training day (Tukey *p* < 0.05) compared to the baseline day (Table 1A). On the testing day, the REM sleep episode numbers also reduced; it was, however, not statistically significant. We did not observe any change in the average REM sleep episode duration on the training and testing days (Table 1B). The SC animals also showed a similar alteration in the sleep-wake architecture after CxFC. There were no changes in wake and NREM sleep amount on the training and testing days (Figures 6A,B). REM sleep, however, significantly decreased (*p* < 0.001, *F*_(2,14)_ = 19.24; *Cohen’s d* = 3.3; power = 0.99 at alpha value 0.05) by 35.8% on training (Tukey *p* < 0.001) and 29.2% on testing (Tukey *p* < 0.01) days compared to the baseline day (Figure 6C). The decrease in REM sleep amount was primarily due to a significant decrease in REM sleep episode numbers (*p* < 0.05, *F*_(2,14)_ = 7.94; *Cohen’s d* = 2.18; power = 0.8 at alpha value 0.05) on training (Tukey *p* < 0.05) as well as on testing days (Tukey *p* < 0.05; Table 1A). REM sleep episode duration, however, did not change (Table 1B).

**Table 1:** Rapid eye movement (REM) sleep episode numbers and duration in non-sleep deprived (NSD), stress control (SC) and sleep-deprived (SD) mice on the baseline, training and testing days of contextual fear conditioning.

Group	Baseline	Training	Testing
**A** REM sleep frequency (Mean ± SEM)			
NSD	18.6 ± 2.99	13.6 ± 1.36*	15.5 ± 2.53
SC	21.8 ± 3.21	15.8 ± 3.18*	15.6 ± 2.20*
SD	23.0 ± 3.65	0	29.80 ± 4.73
**B** REM sleep frequency (Mean ± SEM)			
NSD	1.19 ± 0.22	0.97 ± 0.08	0.96 ± 0.11
SC	1.11 ± 0.14	1.01 ± 0.10	1.09 ± 0.11
SD	1.0 ± 0.16	0	0.82 ± 0.08

*signifies p < 0.05: significant decrease compared to the baseline.

Similar to the NSD and SC mice, the SD animals also exhibited a comparable amount of wake and NREM sleep on baseline and testing days (Figures 6A,B). However, unlike NSD and SC animals, the SD animals did not show decreased REM sleep amount on the testing day (Figure 6C). They exhibited 13.8% more REM sleep on the testing day compared to the baseline day; it was, however, statistically not significant. The NSD and SC animals exhibited less REM sleep, whereas the SD animals showed 60% and 34.7% more REM sleep (*p* < 0.05, *F*_(2,14)_ = 4.06, *Cohen’s d* = 1.48; power = 0.47 at alpha value 0.05) compared to the NSD and SC animals, respectively, on the testing day (Figure 6C). There were no statistical changes in frequency and duration of REM sleep on the testing day in the SD animals (Table 1).

The SD animals were sleep deprived on CxFC training day for 5 h on the slow-moving walking wheel. A total amount of wake, NREM, and REM sleep during deprivation is shown in Figure 6. The SD animals experienced 94.1 ± 4.18% wake, 5.8 ± 4.18% NREM sleep, and no REM sleep during sleep deprivation (Figure 6). These results show that the SD mice were almost awake during the deprivation period.

## Discussion

We have found that short-term sleep deprivation alters the consolidation of contextual fear memory in Swiss mice. The NSD and SC mice exhibited a robust freezing response, but the SD mice showed a reduced freezing response (Figure 5) on the testing day. The reduced freezing response of the SD mice during the entire period on the testing day suggests that they were less fearful from the very beginning itself. Some previous reports suggest that short-term sleep deprivation may alter the consolidation of contextual fear memory in less anxious mice (Graves et al., 2003). Our results are consistent with the previous findings that sleep deprivation impairs the consolidation of contextual fear memory in moderately-anxious Swiss mice.

It has also been proposed that the effects of standard methods of sleep deprivation on memory consolidation could be confounded with stress mediated effects (Cai et al., 2009). The high-intensity light acts as an intense stressor and induces anxiety in rodents (Hale et al., 2006; Bouwknecht et al., 2007; Nathiya and Vanisree, 2010). Therefore, we chose to use high-intensity light as a stressor for the SC group. We did not find memory deficit in the SC animals. The freezing response in the SC animals was comparable to the NSD animals. It is possible that different stressors may induce different levels of anxiety and it would be tough to measure the difference in the amplitude of induced uneasiness by two different stressors. It has been reported that short-term sleep deprivation and brief exposure (30 min) to high-intensity light causes an almost similar fold increase in plasma cortisol level in rodents (Hairston et al., 2001; Ishida et al., 2005). It is also possible that SD mediated, and high-intensity light-mediated increase in the plasma cortisol level may take place at different time points (one may be quick, and the other may be a delayed response). However, these studies do suggest that SD and high-intensity light-mediated induced stress causes similar endocrine changes.

Our result, short-term sleep deprivation induces memory impairment in Swiss mice, is comparable with Graves et al., 2003 findings but is inconsistent with the report of Graves et al. (2003) and Cai et al. (2009). The differences in results could be attributed to: (a) Cai et al. (2009) have used C57BL/6Jx129T2SvEms hybrid mice strain, whereas Graves et al. (2003) have used low anxious C57BL/6J mice and we have used moderately-anxious Swiss mice. C57BL/6Jx129T2SvEms hybrid mice strain has mostly been used in mutant studies (Wood and Anagnostaras, 2011). Hybrid strains demonstrate high anxiousness (Griebel et al., 2000), and it is possible that highly anxious strains may demonstrate more fearful behavior even with gentle handling. (b) Cai et al. (2009) have trained the animal during the late phase of the dark period, whereas Graves et al. (2003) and we have trained the animals during the light phase of the circadian time. The difference in the time of the training period affects learning (Kumar and Jha, 2012), and this could be another reason for the differences in our results.

How sleep benefits the consolidation of contextual fear memory is not known. Some reports suggest that several factors may cause sleep-deprivation mediated cognitive deficits. For example, sleep deprivation alters signaling and activity level of mTORC1, cAMP/PKA, PDE4, GluR1dephosphorylation (which limits receptor insertion into the synaptic membrane), BDNF and pCREB in the hippocampus. These factors are necessarily required for the consolidation of contextual fear memory (Havekes et al., 2007; Vecsey et al., 2009; Hagewoud et al., 2011; Nami et al., 2014; Tudor et al., 2016). We did not ascertain in this study if a change in any of the above mentioned molecular machinery could be the possible reason for sleep-loss-dependent impairment in the consolidation of fearful memory. However, in one of our preliminary studies we have found that a structural gene *formin2*, in the hippocampus, plays a vital role in the regulation of this sleep-dependent consolidation of contextual fear memory in Swiss mice (Qureshi and Jha, 2014).

The consolidation of CxFC memory could be sleep-dependent but may not require augmented sleep. In this study, although contextual fear-conditioning influences sleep architecture, the total amount of wakefulness and NREM sleep (out of TRT) did not change in Swiss mice. Earlier studies have reported that NREM sleep significantly increased in less anxious mice (Hellman and Abel, 2007), but significantly decreased in more anxious mice (Sanford et al., 2003b) during the 24 h post-conditioning period. It suggests that fear conditioning may influence sleep differently in different strains. Our results here demonstrate that CxFC did not alter NREM sleep in moderately anxious mice. It is, however, not known if the expression of NREM sleep after fear-conditioning would have any correlation with the varying levels of anxiousness in different strains. It would require more studies to establish this relationship. Our findings at least indicate that there might be some association between different anxiety levels and variations in NREM sleep expression after fear conditioning.

Furthermore, REM sleep decreased significantly in the NSD and SC animals but remained unaltered in SD animals. REM sleep is considered to be the sensitive index of fear-conditioning (Jha et al., 2005a). It decreases significantly after cued-fear conditioning (Sanford et al., 2001; Jha et al., 2005a; Kumar and Jha, 2012, 2017), passive avoidance task (Mavanji et al., 2003) and contextual fear conditioning (Sanford et al., 2003b). Our results that REM sleep significantly decreased after CxFC even in moderately-anxious mice are consistent with these reports. The decrease in the REM sleep amount was primarily due to the decrease in frequency of REM sleep. Further, we have recently reported that REM sleep decreases exclusively in consolidated memory groups but not in impaired memory groups (Kumar and Jha, 2017). Why REM sleep decreases after fear conditioning is not known. No change in REM sleep amount in SD animals suggests that it could be associated with the consolidation of fearful memory. We have reported earlier that if the contextually fear-conditioned animals sleep in fearful context, they exhibit reduced REM sleep. Further, REM sleep increases when the animals sleep in a neutral novel context (Pawlyk et al., 2005). Also, REM sleep increases with fearful memory extinction but decreases after fear conditioning (Wellman et al., 2008). Similarly, REM sleep decreases with considerable fear-training with inescapable shock but increases with escapable shock (Yang et al., 2011). The animals trained with inescapable shock demonstrate stronger fear memory trace during testing than the animals trained with escapable shock (Mineka et al., 1984). All these studies suggest that decreased REM sleep and the consolidation of fearful memory could be interrelated.

How fear-conditioning alters REM sleep is yet again not known. However, REM sleep is usually altered by fearful stressors and contexts (Jha et al., 2005a; Pawlyk et al., 2005). It appears that distinct neural networks modulate fear memory and fear-induced changes in REM sleep. Corticotropin-releasing factor (CRF) in the basolateral amygdala (BLA) seems to be involved in the regulation of stress-induced alterations in REM sleep, but not in the modulation of fear behavior (Wellman et al., 2013). Interestingly, bilateral inactivation of CRF1 receptors into the BLA before training blocked fear-induced reductions in REM sleep on training and testing days, but not the freezing behavior (Wellman et al., 2013). It implies that fear memory can be stabilized even without REM sleep reduction. In our study, REM sleep decreased in the consolidated memory groups but did not change in the impaired memory group, indicating that the consolidation of fear memory and reduced REM sleep could be linked but may not have an obligatory association.

In summary, our results show that sleep soon after training plays an essential role in the consolidation of contextual fear memory. A short-term sleep deprivation soon after contextual fear training impaired fear memory. Since the high-intensity light stressor did not impair fear memory, the effect of sleep deprivation on memory could be attributed to sleep loss. Further, NREM sleep did not change after fear conditioning, but REM sleep significantly decreased in moderately-anxious Swiss mice. Our study, along with others, indicates that there may be some correlation between the varying anxiety levels and corresponding changes in NREM sleep amount. It, however, requires future studies to establish a definite correlation. We find that REM sleep is reduced after fear conditioning, but an in-depth study is warranted to say if this reduction is fear-induced or an obligatory phenomenon required for stabilization of fearful memory.

## Author Contributions

MFQ: performed experiments, generated and analyzed data and prepared the initial draft of the manuscript. SKJ: conceived the idea and designed the work, analyzed the data and finalized the manuscript.

## Conflict of Interest Statement

The authors declare that the research was conducted in the absence of any commercial or financial relationships that could be construed as a potential conflict of interest.
